# Transcranial Direct Current Stimulation in Treatment of Child Neuropsychiatric Disorders: Ethical Considerations

**DOI:** 10.3389/fnhum.2022.842013

**Published:** 2022-07-08

**Authors:** Narong Auvichayapat, Paradee Auvichayapat

**Affiliations:** ^1^Noninvasive Brain Stimulation Research Group of Thailand, Khon Kaen University, Khon Kaen, Thailand; ^2^Division of Pediatric Neurology, Department of Pediatrics, Khon Kaen University, Khon Kaen, Thailand; ^3^Department of Physiology, Faculty of Medicine, Khon Kaen University, Khon Kaen, Thailand

**Keywords:** transcranial direct current stimulation (tDCS), ethical considerations, child neuropsychiatric disorders, noninvasive brain stimulation, pediatric neurology

## Abstract

Transcranial direct current stimulation (tDCS) is a noninvasive electrical stimulation performed using low electric currents passing through two electrodes. The provided current passes from the anode to the cathode and induces electric fields in the surface neurons. It then modulates synaptic plasticity and finally changes cortical excitability or improves clinical outcomes, which outlast after a duration of stimulation. Meta-analyses have supported the beneficial effects of tDCS treatments in child neuropsychiatric disorders. However, the study of vulnerable children remains controversial and is a great deal for ethical considerations. Because the developing brain has some important physiological differences from the matured brain, specifically less γ-aminobutyric acid (GABA)ergic inhibition and more myelination, the opportunity to modify neurological disorders to be close to the normal level in childhood after tDCS is likely to be higher than in adults. In contrast, these physiological differences may result in unexpected excitability in children's brains and were criticized to have an unsafe effect, specifically seizures, which is a serious adverse events. As mentioned above, using tDCS in children appears to be a double-edged sword and should be ethically considered prior to wide use. Assessing between benefits of tDCS treatment within the golden period of brain development and the risk of seizure provocation is important. Thus, this perspective article is aimed to exhibit broad concepts about the developing brain, tDCS in children, pathophysiology of neuropsychiatric disorders and tDCS beneficence, tDCS safety and tolerability in children, and missing good opportunities or taking risks in tDCS.

## Introduction

The utilization of an electrical stimulation treatment for an individual with the neuropsychiatric disorder is not a new therapeutic method. It was first discovered in the Greco-Roman era around 1 B.C. using an electric fish to be an electrical source for treating severe headaches. Treatments using electrical stimulation have evolved over time. Nowadays, there are many pieces of evidence that show the benefits of electrical stimulations on neuropsychiatric disorders. Transcranial direct current stimulation (tDCS) is a noninvasive electrical stimulation that uses a constantly low direct current delivered *via* the electrodes on the patient's head. The tDCS mechanism emerges from synaptic plasticity modulation. Despite the neuronal modulation effect of the tDCS, there are some criticisms about its unsafe effect in children, such as seizures, which is a serious adverse events. Accordingly, a judgment of tDCS is necessary before using it in child neuropsychiatry disorders, such as autism spectrum disorder (ASD), attention deficit hyperactivity disorder (ADHD), childhood epilepsy, child motor dysfunction, and pediatric migraine. The broad concept of the developing brain, tDCS in children, pathophysiology of neuropsychiatric disorders and tDCS beneficence, and tDCS safety and tolerability in children should be considered when discussing missing good opportunities or taking risks on tDCS.

## Developing Brain in the Overview

Childhood is the golden period of life where many new experiences are gained. Nature allows children to learn new skills, such as language, learning, thinking, arithmetic, and music quickly and continuously. As they mature, their learning ability slows down and becomes more difficult (Kolb et al., 2017).

The difference in learning ability between children and adults can be clarified at the neurological level. Neural development in the fetal period consists of the development of neuronal cells, beginning with a nuclear migration at week 4 and then dividing and moving into all six cortical layers by week 28. After childbirth, human brains grow five times in size, with the total number of neurons virtually unchanged. Most of the postnatal growth includes axon myelination and synaptogenesis (Budday et al., 2015).

### Synaptogenesis

Humans create more synapses than mice, which helps the human to brain learn and adapt more. New neural connection is generated continuously, and the development of synaptic pruning and modern neural connections are delivered persistently. Neurons do not have the ability to synapse at the beginning, but this ability is obtained through the exposure to astrocytes. Synaptogenesis consists of three stages, which are as follows: (1) immature synapses are formed between the axon and dendrite, (2) the synapse matures and develops to an active state, and (3) the synaptic number is reduced by synaptic pruning to refine the neuronal connections within the circuit (Budday et al., 2015). The synaptic connections start before week 7 but occur mainly after birth, begin in the visual cortex, and occur most rapidly between 2 and 4 months of age. Almost 50% of those synapses were eliminated by synaptic pruning at about 8 months of age. Synaptogenesis in other brain regions, such as the frontal cortex, also sequentially develops after 15 months of age. Excess synaptic connections at the beginning of life are valuable for children to gain experiences as much as possible. Only the most important neuronal circuits are selected for survival and growth by trimming the inactive synapses depends on one's experience. The number of synapses in the prefrontal cortex remained higher than usual until the early adulthood. Recent studies reveal that producing and refining synaptic spines continue until approximately 30 years of age (Limburg et al., 2016).

Synaptic plasticity is the change in strength and rate of neuronal signal in synapses according to the activity performed by the individual. It is the mechanism that plays a significant role during synaptic connection. Increased synaptic strength results from long-term potentiation (LTP), whereas reduced synaptic strength results from long-term depression (LTD). Both LTP and LTD can be modulated by different forms of tDCS. Furthermore, the synaptic plasticity increases during child development due to less γ-aminobutyric acid (GABA)ergic inhibition in childhood as compared to adults (Hameed et al., 2017). Functional magnetic resonance imaging (fMRI) or diffuse tensor imaging can detect changes in brain structures. For example, developmental changes are observed in the corpus callosum, a structure that connects both hemispheres of the brain. This link allows the exchange of data integration. In addition, there is evidence that the corpus callosum becomes larger in the early postnatal period by increasing both size and number of nerve fibers. Myelination of nerve fibers begins at approximately 6 months of age and increases gradually. Continuous myelination of individual brain regions of the white matter is critical for cognitive development. Previous studies found that language development and increased memory capacity progress with continued myelin formation. Intellectual and motor skills also develop according to the structure and function of the motor cortex (Limburg et al., 2016). The new neural connections, plasticity, and myelination are the most important preconditions for learning in the developing brain; thus, the use of tDCS to facilitate these effects seems to be more effective in developing than in the mature brain. Synaptogenesis causes brain connectivity, as shown in the developmental period of important pathways summarized in Table 1.

**Table 1:** Developmental period of brain connectivity.

**Brain connectivity**	**Developmental period**
**Interhemispheric connections:**	
Corpus callosum	Week 8 to 20
Commissural connections	Week 28 to 32
**Intra-hemispheric connections**:	
Thalamo-cortical connections	Weeks 22 to 27
Cortico-cortical association fibers	Week 28 to 32
**Short horizontal connections**	
Cortical gray matter and subcortical white matter	Week 32 to 47

### Synaptic Pruning

In humans, synaptic pruning, the elimination of synapses, begins at birth and continues to develop until the end of the adolescence. Synaptic pruning is influenced by environmental factors and is widely thought to represent learning.

## tDCS in Children

The tDCS is a non-invasive form of brain stimulation that uses a low electric current passing through two electrodes (anode and cathode). This may change the regional cortical excitability to a state of excitation or inhibition. The supplied current passes from the anode to the cathode induces the modulation of electrical fields in surface neurons to change the neuronal excitability and induces spontaneous firing in axons (Ciechanski and Kirton, 2016). The axonal firing rate may result in interneuronal synaptic modulation that makes both anodal and cathodal tDCS outlast the duration of the stimulation. Various neurotransmitters, such as amphetamines (catecholamine reuptake blockers), dopamine, and GABA, have been reported to enhance and prolong the cortical hyperexcitability following anodal tDCS. Obtaining early the serotonin reuptake inhibitor enhances cortical excitability following anodal tDCS and shifts the inhibitor toward excitatory effects after cathodal tDCS. The magnetic resonance spectroscopic study has presented changes in neurotransmitter and metabolite concentrations within the brain. Cortical neurochemistry changes occurred under the target electrode and affected the GABA, glutamine, and glutamate concentrations. After anodal tDCS, the decrease in GABA is observed following increases in combined glutamine and glutamate, as well as myo-inositol concentrations. Little has been studied about cathodal tDCS in regard to neurochemical changes; however, a prior study proposed that cathodal tDCS decreases the concentration of GABA and combined glutamate and glutamine. Resting-state fMRI studies revealed that both anodal and cathodal stimulations may influence default mode networks (Ciechanski and Kirton, 2016). The physiological actions of tDCS in children's brains differ from that of adults, probably due to head anatomy, such as head size, skull thickening, and cerebrospinal fluid (CSF) volume, as well as brain physiology, such as the different relative amounts of neurotransmitters and neurotrophies. GABA can have excitatory actions during development, particularly in the early developmental period. These factors can also result in the alteration of the brain's responsiveness to tDCS and must be considered carefully when applying tDCS to children (Friel et al., 2016). The impacts of cathodal stimulation are nonlinear, with incitement creating distinctive effects, and possibly have the opposite effects in children. A previous study was conducted by applying either 2 mA anodal, 2 mA cathodal, or 1 mA cathodal tDCS to young adult participants. A 2-mA cathodal stimulation appeared to mimic the effects of 2-mA anodal tDCS by increasing the cortical excitability, as seen by larger motor-evoked potential (MEP) amplitudes, whereas 1 mA cathodal tDCS decreased MEP amplitudes. These findings suggest that the traditional anodal-excitatory/cathodal inhibitory model may be more complex and requires additional study in children (Ciechanski and Kirton, 2016). Since the anatomical and physiological properties are different between children and adults as mentioned, the bioavailability of the current or the electric field strength experienced by the cortex may differ significantly. Depending on numerous factors, the children's brain cortex may experience double intensities of the electric field as compared to adults while receiving the same current. Therefore, 1 mA cathodal current applied to children may actually have the same effect as that of 2 mA in adults (Ciechanski and Kirton, 2016).

## Pathophysiology of Neuropsychiatric Disorders and Benefits of tDCS

In the human cerebral cortex, there are 10–20 billion neurons. The cooperation of these neurons depends on the effectiveness of the synapse, which has approximately 60 trillion synapses, hundreds of times more than neurons (Gordon, 1998). These neurons communicate with each other for proper functioning, such as learning, memory, and motor movement via multiple synapses. An important and interesting property of the synapses is synaptic plasticity, which refers to the flexibility or functional modification of a synapse by modifying the strength or efficiency of synaptic transmissions. To maintain the proper functioning of the nervous system, synaptic plasticity is either increased by LTP or decreased by LTD to adjust the neuronal flexibility (Citri and Malenka, 2008). The synaptic plasticity also plays an important role in the early development of neural circuits. The defects in synaptic development, structure, and plasticity have been hypothesized to be the underlying causes that alter the neuronal function in complex child neuropsychiatric disorders (Wang et al., 2018).

## Autism Spectrum Disorder

Autism spectrum disorder is the abnormal formation of synapses and dendritic spines (Persico and Bourgeron, 2006), impairment of synaptic pruning (Kim et al., 2017), and lack of synaptic plasticity (Hansel, 2019). There remains no curative treatment for autistic individuals. Autism therapies mainly aim to lessen the deficits and abnormal behaviors, as well as to increase the quality of life. The result of autism treatment is poor; only 1–2% of patients with autism can live normally, with most patients with autism remaining dependent on caregivers. A lifelong condition in ASD consumes long-term societal and familial costs; total costs per year for children with ASD and medical expenditures are 4–6 times more noteworthy than for individuals without ASD (Shimabukuro et al., 2008). As noted above, ASD pathophysiology is the abnormal formation of synapses and dendritic spines and lack of synaptic plasticity (Hansel, 2019). The tDCS was considered in regulating synaptic plasticity (Hameed et al., 2017), increasing glial cell activity and synaptogenesis (Auvichayapat et al., 2020), and promoting cortical meta-plasticity (Monai et al., 2016) and early gene expression (Moriwaki et al., 1995). It is therefore postulated that tDCS is a method for treating ASD pathology (Levy et al., 2009) with several studies supporting the potential benefit of tDCS in individuals with ASD (Schneider and Hopp, 2011; Amatachaya et al., 2014, 2015; D'Urso et al., 2015; Gómez et al., 2017; Esse Wilson et al., 2018; Rothärmel et al., 2019; Auvichayapat et al., 2020; Hadoush et al., 2020). Moreover, in a recent meta-analysis of three children with ASD, 52 participants showed significant improvements in three subdomains, such as the Autism Treatment Evaluation Checklist (ATEC) social subscale score (standardized mean difference [SMD] = −0.68, 95% CI (−1.05, −0.32), *p* < 0.001), health and behavioral subscale score (SMD = −0.66, 95% CI (−1.02, −0.30), *p* < 0.001), and the total score (SMD = −0.97, 95% CI (−1.58, −0.36), *p* < 0.001), while the heterogeneity test was not significant in all cases (García-González et al., 2021).

## Attention Deficit Hyperactivity Disorder

Attention deficit hyperactivity disorder is another common neurodevelopmental disorder with unclear pathophysiology. The mechanisms proposed that factors associated with abnormal neurotransmitter imbalances include decreased dopamine and noradrenaline (Sharma and Couture, 2014). According to the mechanism of tDCS in neuromodulation and alteration in some neurotransmitters, tDCS has therefore been used in ADHD treatment. A previous systematic review and meta-analysis of 45 studies indicated that tDCS had significant overall effect of inhibitory control (g = 0.23 [SE = 0.046, 95% CI = 0.14–0.32], z = 5.03, *p* < 0.00001). The model also revealed a low-to-moderate amount of residual heterogeneity [Quantitative easing (QE)(74) = 116.4, *p* = 0.001, 95% CI = 11.8–52.8%, Akaike information criteria (AIC) = 76.6]. However, the between-studies variability (g = 0.21) was moderate (g = 0.21), which was due to the locations of target and return electrode placement and the tasks used in each study (Schroeder et al., 2020).

## Childhood Epilepsy

Childhood epilepsy is a disease caused by the lack of inhibition in neural circuits (disinhibition), causing abnormal electrical discharge merging in multiple neurons (synchronization) (Rho and Stafstrom, 2006; Lang, 2016). There is evidence that neuronal loss is initiated by prolonged convulsion and may contribute to improper synaptogenesis in the model of status epilepticus (Morimoto et al., 2004). However, neuronal plasticity can increase sensitivity or modulate the inhibitory system. It also causes a disinhibition process that becomes a long-term change and promotes epileptic attacks. The misfortune of particular sorts of interneurons, changes in GABA receptor configuration, and/or decrease in dendritic inhibition could contribute to spontaneous seizures (Morimoto et al., 2004). tDCS has demonstrated guaranteed results in children with focal refractory epilepsy. A case series also showed decreased seizure frequency after applying 0.3–0.7 mA anodal tDCS over the posterior temporal cortex with the reference electrodes over the parietal cortex for 20–40 min at maximum of 15 sessions (Shelyakin et al., 2001). Another randomized controlled trial (RCT) applying 1 mA cathodal tDCS over the seizure focus for 20 min reported a significant reduction in epileptiform discharges for 2 days (Auvichayapat et al., 2013). In addition, 2 mA cathodal tDCS for 20 min at five consecutive sessions, performed above the left primary motor cortex with the anodal electrode placed on the right shoulder, showed a significantly reduced seizure frequency in the active tDCS group as compared to the sham group from the first day to the 4th week after tDCS stimulation in Lennox-Gastaut syndrome (Auvichayapat et al., 2016). Another case report showed decreased epileptiform discharges after 5 consecutive days of 2 mA cathodal tDCS over the epileptic focus for 2 weeks (Yook et al., 2011).

## Child Motor Dysfunction

Child motor dysfunction is a physical disability caused by brain damage before birth, during birth, or in the childhood period. The pathophysiology of child motor dysfunction consists of the interruption of the brain oxygen supply. It is considered as the main causal factor of neuronal cell death, causing abnormal motor function at a later time. Therefore, synaptic plasticity is not a major cause in terms of pathophysiology as found in ASD and epilepsy. Motor pieces of training, especially task-specific repetition, result in increased synaptic plasticity that can augment the capacity of the intact portion of the brain and can reorganize the new neuronal circuits to replace the damaged brain functions (Marret et al., 2013).

The most common cause of pediatric motor dysfunction is cerebral palsy (CP), which affects 2.5 out of 1,000 newborns (Saleem et al., 2019). The prevalence of pediatric stroke has expanded by roughly 35% between 1990 and 2013 (Krishnamurthi et al., 2015) and has been one of the top 10 causes of childhood mortality (Mittal et al., 2015). The neurological rehabilitation of pediatric motor disorders uses a motor-learning approach primarily to improve motor behavior and promote performance (Saleem et al., 2019). Meta-analyses have concluded that there is strong support for the beneficial effects of tDCS in child motor dysfunction, specifically a systematic review and meta-analysis in 23 motor disorder studies, with 373 participants with CP; 33 participants with dystonia; involuntary movements and delayed neuromotor development. The mean study sample size was 16 participants (range 1–56) with ages ranging from 4 to 21 years. The study revealed the effectiveness of tDCS in improving gait velocity {Mean Difference (MD) = 0.23; 95% CI [0.13, 0.34]; *p* < 0.0005}, stride length (MD = 0.10; 95% CI [0.05, 0.15]; *p* < 0.0005), and cadence (MD = 15.7; 95% CI [9.72, 21.68]; *p* < 0.0005) (Saleem et al., 2019). Moreover, the meta-analysis of 10 studies, consisting of 306 participants with CP, showed noteworthy changes in all upper limb capacities (SMDs between 0.94 and 1.83; *p* = 0.0001) and balance (SMDs between −0.48 and 0.83; *p* < 0.05) (Elbanna et al., 2019). A systematic review and meta-analysis of tDCS treatment studies in children with aphasia, which included eight tDCS studies with 140 participants, revealed pooled SMD of 0.395 (*p* < 0.001) in favor of tDCS. Little is known about the mechanism of tDCS that can reduce spasticity; however, a previous study showed an increase in some brain metabolites after tDCS in CP participants and postulated that it might be impacted by neuronal connections (Auvichayapat et al., 2017).

Even with such intense tDCS studies in child motor dysfunction, there were no serious adverse events found. The meta-analyses showed that the most common adverse effects in the active tDCS group were tingling (17%), discomfort (8%), itching (7%), and skin redness (4%) (Saleem et al., 2019).

## Pediatric Migraine

Studies using tDCS in the treatment of migraines and pain in children have not been published. However, with the increasing number of tDCS use in adults, it can be hypothesized that the use of this technique is effective in children. Evidence of cathodal tDCS over the visual cortex showed a significant improvement in pain. No serious side effects were reported. These effects are important in the context of the pediatric population (Brighina et al., 2019).

Transcranial direct current stimulation safety' clinical outcomes and adverse events in children with neuropsychiatric disorders are summarized in Table 2.

**Table 2:** Summarized tDCS' clinical outcomes and adverse events in children with neuropsychiatric disorders.

**Authors (Year)**	**Research type**	**Subject**	**Number**	**Age**	**Protocol**			**Outcome**	**Result**	**Adverse event**
					**Session**	**Anodal**	**Cathodal**			
Schneider and Hopp (2011)	Before and after study	Autism Spectrum Disorders	10	6-21	Single dose tDCS, 30 min	Left DLPFC	Right supraorbital region	Bilingual aphasia test	Significant increase mean syntax scores 247%	None
Amatachaya et al. (2014)	centerRCT and cross over	Autism Spectrum Disorders	20	5-8	1 mA, 5 sessions tDCS 20 min	Left DLPFC	Right shoulder	CARS, ATEC, CGAS, CGI-I	Significant decreased CARS, ATEC, CGAS, CGI-I	None
Amatachaya et al. (2015)	RCT and cross over	Autism Spectrum Disorders	20	5-8	1 mA, Single dose tDCS, 20 min	Left DLPFC	Right shoulder	Peak alpha frequency	Significant Increase peak alpha frequency	None
Gómez et al. (2017)	RCT	Autism Spectrum Disorders	9	5-10	1 mA, 20 sessions tDCS, 20 min	Right arm	Left DLPFC	ATEC, ADI-R, ABC, EEG	Significant decreased ATEC, ADI-R, ABC Increase functional connectivity in alpha, beta, and gamma frequency	Mild
D'Urso et al. (2015)	Before and after study	Autism Spectrum Disorders	12	18-26	1.5 mA, 10 sessions tDCS, 20 min	Right arm	Left DLPFC	ABC	Significant decreased ABC	None
Esse Wilson et al. (2018)	RCT	Autism Spectrum Disorders	6	18-58	2 mA, 2 sessions tDCS (1-week interval), 30 min	Right temporoparietal junction	Left deltoid	ATEC	Significant decreased ATEC	Mild
Rothärmel et al. (2019)	Before and after study	Autism Spectrum Disorders	8	20-28	2 mA, 10 sessions tDCS, 15 min	Right supraorbital area	Left DLPFC	1.Executive function: SCWT, TMT-A/B, mWCST, VFT 2. Behavioral dysexecutive syndrome: BDSI 3. Repetitive behaviors: RRB	1.Executive function: Significant decreased initial time TMT-A/B 3. Repetitive behaviors: Significant decreased RRB	Mild
Hadoush et al. (2020)	RCT	Autism Spectrum Disorders	50	4-14	10 sessions tDCS, 20 min	Left and Right prefrontal cortex	Right and left supraorbital areas	ATEC	Significant decreased ATEC	None
Auvichayapat et al. (2020)	Before and after study	Autism Spectrum Disorders	10	5-8	1 mA, 5 sessions tDCS, 20 min	Left DLPFC	Right shoulder	1. ATEC 2. NAA, Cho, mI, Glx	1. Significant decreased ATEC 2. Significant increased NAA and mI in the left DLPFC and locus coeruleus	None
Shelyakin et al. (2001)	Case series	Childhood epilepsy	18	4-8	<15 sessions, 20–40 min	Depend on brain pathology	Depend on brain pathology	Epileptic discharge	Significant decreased epileptic discharge	N/A
Auvichayapat et al. (2013)	RCT	Refractory focal childhood epilepsy	36	6-15	1 mA, single-dose tDCS, 20 min	Contralateral shoulder	Epileptogenic focus by EEG	1. Epileptic discharge frequency 2. Seizure frequency	1. Significant reductions in epileptic discharge frequency 2. Decrease in seizure frequency	Transient erythematous rash under the reference electrode
Auvichayapat et al. (2016)	RCT	Lennox-Gastaut	22	3-9	2 mA, 5 sessions, 20 min	Right shoulder	Left primary motor cortex	1. Clinical seizure 2. Epileptiform discharges	1. Significant reduction in seizure frequency 2. Significant reduction in epileptiform discharges	Transient superficial skin burns
Yook et al. (2011)	Case report	Childhood epilepsy due to focal cortical dysplasia	1	11	2 mA, 10 sessions, 20 min	Left orbit	Between P4 and T4	Seizure frequency and duration	Great reduction of seizure frequency and duration	N/A

*ABC, Aberrant Behavior Checklist; ADI-R, Autism Diagnostic Interview Revised; ATEC, Autism Treatment Evaluation Checklist; BDSI, Behavioral Dysexecutive Syndrome Inventory; CARS, Childhood Autism Rating Scale; CGAS, Children's Global Assessment Scale; CGI-I, Clinical Global Impression Improvement; Cho, Choline; DLPFC, Dorsolateral prefrontal cortex; EEG, Electro-encephalography; Glx, Glutamine combined glutamate; mA, Milli Ampere; mI, Myoinositol; mWCST, Modified Wisconsin Card Sorting Test; N/A, Not applicable; NAA, N-acetyl aspartate; RCT, Randomized controlled trial; RRB, Repetitive and Restricted Behavior Scale; SCWT, Stroop Color – Word test; tDCS, Transcranial direct current stimulation; TMT-A/B, Trail Making Test A and B; VFT, Verbal Fluency Test*.

## tDCS and Tolerability in Children

Although many tDCS studies have evidence supporting their efficacy in child neuropsychiatric disorders, the safety and tolerability in children should still be considered. A recent systematic review of tDCS safety in children and adolescents was conducted with data collection from the beginning of the database to November 2019. The data included 12 articles, 156 children, and 864 active tDCS sessions (0.5–2 mA) with a total of 303 h of stimulation, aged 6–17 (mean 10.75) (Buchanan et al., 2021). The result showed no serious adverse events in all 12 studies. In total, 83% of the studies reported adverse events and tolerability. The questionnaires used in those studies were following the Poreisz Security Safety Review (Poreisz et al., 2007). The review investigated side effects, such as burning, itching, tingling, pain, rash, blisters, fatigue, insomnia, nausea, mood changes, and difficulty concentrating. There were neither neuroimaging adverse events nor clinical adverse events. Psychiatric adverse events were reported in only 1 of 12 studies described by 42.9% mood change and 35.7% irritability (Andrade et al., 2014). Cognitive adverse events, mostly fatigue for 14–32%, were reported in 4 of 12 studies; trouble concentrating was reported for 14–20% in 2 of 12 studies. Physical adverse events were the most frequent in children, with 25–54% of tingling and itching in 5 of 12 studies; 14–31% burning in 3 of 12 studies; 14–25% headache in 2 of 12 studies; 7–11% pain under the electrode placement in 2 of 12 studies; and 3% skin rash in 1 of 12 studies. Almost all studies had no dropout participants except one study with an 8% dropout rate. No seizure has been reported during tDCS sessions (Bikson et al., 2009). However, a case report of a 4-year-old boy who developed seizures at 4 h after the third session of tDCS stimulation was presented. This participant had underlying diseases of spastic tetraparesis and uncontrollable epilepsy prior to the tDCS treatment. The patient was enrolled in an outpatient tDCS study because he wanted to improve his upper limb function and reduce his spasticity (Ekici, 2015). Although tDCS has a very low risk for children with seizures, tDCS could possibly provoke seizure as mentioned above. Therefore, tDCS treatment should be taken with caution in a patient with uncontrollable or unstable epilepsy defined as more than one seizure per month or a recent change in seizure frequency or medication (Gordon, 1998; Friel et al., 2016).

## Discussion

Since the GABAergic inhibition in children is lesser than the adults' brains, the neuronal plasticity in children is certainly exceeding (Palm et al., 2016). According to the evidence, tDCS modulates neurons via the GABAergic inhibitory to result in two possible ways in children. Firstly, it will facilitate the process of neuronal plasticity that will benefit children with neuropsychiatric diseases as mentioned above. Secondly, because the degree of excitability cannot be predicted in such an immature brain, it might bring serious adverse effects, such as seizures. However, to the best of our knowledge, the seizure happened in only one patient who has underlying uncontrollable epilepsy (Hameed et al., 2017). According to the tDCS human experimental trial, the serious adverse effect has not yet been reported as noted above (Poreisz et al., 2007; Andrade et al., 2014; Buchanan et al., 2021). The other important point which has been criticized is that the tDCS device has not been approved by the Food and Drug Administration (FDA) Neuromodec, 2021. It does not cruel for the FDA to have made a formal choice on adequacy or security of tDCS but it implies that the FDA has not assessed and affirmed an application from a company yet Neuromodec, 2021. Altogether, tDCS is a plausible therapy in children with neuropsychiatric disorders, particularly in abnormal synaptic and neuronal inhibitory function, while taking a very low risk of adverse effect. In addition, tDCS is more advantageous than other noninvasive brain stimulation i.e., TMS in potentially performed home therapy. Children with neuropsychiatric disorders who have not been treated with tDCS could possibly lose the opportunity, which creates more incumbrance in the future than in children who have been taking risks from tDCS treatment.

## Conclusion

Despite children belonging to a vulnerable group for the tDCS studies, child brain physiological function is another aspect for consideration. Childhood is the golden period of neuronal network restoration that can be facilitated by tDCS. While letting the disease progress to adulthood, the efficiency of neurological recovery would decline; thus, the impact of tDCS would not yield as great benefit as in the youth. Since children have no right to choose for themselves, good opportunity is lost every year as they grow up. When they become adults, having the authority to choose, the treatment would not provide as many advantages. Therefore, tDCS use in children with neuropsychiatric disorders should be ethically considered from a new perspective.

## Data Availability Statement

The original contributions presented in the study are included in the article/supplementary material, further inquiries can be directed to the corresponding author.

## Author Contributions

PA: manuscript writing on neurophysiology. NA: manuscript writing on child neurology. Both authors contributed to the article and approved the submitted version.

## Funding

This work was supported by a Research Assistantship (AS63206) Faculty of Medicine, Khon Kaen University, Khon Kaen, Thailand.

## Conflict of Interest

The authors declare that the research was conducted in the absence of any commercial or financial relationships that could be construed as a potential conflict of interest.

## Publisher's Note

All claims expressed in this article are solely those of the authors and do not necessarily represent those of their affiliated organizations, or those of the publisher, the editors and the reviewers. Any product that may be evaluated in this article, or claim that may be made by its manufacturer, is not guaranteed or endorsed by the publisher.
